# Plant Regeneration Trait Syndromes, Tradeoffs, and Linkages to Adult Abundance for Native and Exotic Grassland Plants

**DOI:** 10.1002/ece3.72143

**Published:** 2025-09-10

**Authors:** Mandy L. Slate, Phil G. Hahn, Yvette K. Ortega, Marisa Mancillas, Christoph Rosche, Dean E. Pearson

**Affiliations:** ^1^ Rocky Mountain Research Station USDA Forest Service Missoula Montana USA; ^2^ Entomology and Nematology Department University of Florida Gainesville Florida USA; ^3^ Division of Biological Sciences University of Montana Missoula Montana USA; ^4^ Animal and Range Sciences New Mexico State University Las Cruces New Mexico USA; ^5^ German Centre for Integrative Biodiversity Research (iDiv) Leipzig Germany; ^6^ Institute of Biology/Geobotany and Botanical Garden Martin Luther University Halle‐Wittenberg Halle (Saale) Germany

**Keywords:** aridity gradient, community assembly, invasive exotic plant, recruitment, seed and seedling traits, trait‐by‐environment

## Abstract

Recruitment is the most sensitive plant life stage to environmental filters. Yet, most research linking functional traits to environmental filters has focused on adult plants with little known about early plant traits, their interactions with environmental filters, or their relation to species abundance. Likewise, how such relationships might vary between native and exotic species or influence plant invasion outcomes is unclear. We quantified regeneration traits for 12 native and 12 exotic (naturalized and invasive) forbs and evaluated trait relationships and their associations with species abundance across an environmental gradient in semi‐arid grasslands. Species differentiated along two orthogonal trait axes suggestive of two distinct trait syndromes. The first trait syndrome, likely associated with competitive ability, was correlated with seed mass and growth‐related seedling traits. Conversely, the second trait syndrome revealed a tradeoff between traits related to development and growth and traits related to resource management. This syndrome may reflect different approaches for seedling stress tolerance and avoidance. Neither trait syndromes nor mean trait values differed between native and exotic species, whether exotics were invasive or naturalized. Two traits and one trait syndrome were significantly associated with adult species abundance on the landscape. First, species with faster seedling maturation were generally more abundant. Naturalized exotic species with lower specific leaf area were also more abundant, suggesting a possible link between lower specific leaf area and greater drought survival. Abundance of native and invasive exotic species was greater for taxa with faster development and growth and thin, carbon‐rich leaves, traits associated with stress avoidance. Importantly, the greater abundance of invasive exotics over other taxa was not accounted for by differences in regeneration traits. Evidence of regeneration trait syndromes and tradeoffs points to important selective forces shaping early plant life‐history strategies. Linkages between some of these traits and adult plant abundance also suggest a significant role in recruitment success. Better elucidating these traits and their connections to species abundance—particularly across life stages—can help improve our understanding of plant community assembly.

## Graphical abstract

Our study is important because it identifies new connections between plant regeneration traits and grassland forb abundance that imply strong selection on early plant traits and present new opportunities for evaluating plant regeneration trait convergence and adaptation. Associations reported here provide a deeper understanding of the selection challenges seedlings face and will significantly improve our ability to connect plant regeneration to more applied aspects of plant ecology such as restoration, conservation, and the impacts of changing climate on these efforts.

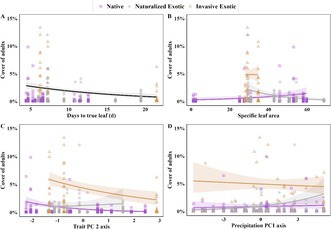

## 1 Introduction

Community assembly theory is based on the premise that the composition and relative abundance of species within a community is determined by how species traits interact with a series of abiotic and biotic filters (Keddy 1992; Weiher et al. 1998). Over the past few decades, ecologists have increasingly relied on trait‐based approaches to understand such ecological variation across plant species within and across communities (e.g., Poorter and Bongers 2006; Cornwell and Ackerly 2009). These functional trait approaches have been instrumental in identifying how unique ecological strategies relate to species abundance (e.g., Wright and Westoby 1999; Bernard‐Verdier et al. 2012; Kunstler et al. 2016). However, most research evaluating linkages between plant functional traits and species abundance has focused on traits of adult plants (e.g., Cornwell and Ackerly 2010; Soudzilovskaia et al. 2013; Yan et al. 2013) and it is less clear how the same linkages apply to regeneration traits, traits related to seed germination and seedling growth (*sensu* Larson and Funk 2016).

Seeds, seedlings, and adults possess many distinct ontogenically based traits, with seedlings being much more sensitive to environmental conditions than adults (Mitchell and Bakker 2014; Zirbel and Brudvig 2020; Havrilla et al. 2021; Nagy et al. 2024). Importantly, transitions from seed to seedling (germination and emergence) and seedling to juvenile (recruitment) are among the most sensitive of plant life‐history stages, with success across each transition impacting adult plant abundance and distribution (e.g., Poorter 2007; Stampfli and Zeiter 2008; James et al. 2011; Larson et al. 2015; Garbowski et al. 2021; Shackelford et al. 2021). However, despite the recognized vulnerability of seed and seedling stages, surprisingly little is known about how associated regeneration traits align into trait syndromes (i.e., are positively correlated), reflect ecological tradeoffs (i.e., are negatively correlated), and relate to adult plant abundance in natural systems.

Covariation and tradeoffs among regeneration traits, links between regeneration traits and key functions, and the tight coordination of these strategies with environmental conditions are thought to be maximized for plant survival (e.g., Schupp 1995; Muscarella et al. 2013; Harrison and LaForgia 2019). Resource economic spectra for adult plants (e.g., Leaf Economic Spectrum; Lambers and Poorter 1992; Reich 2014) are often centered around the importance of traits and trait relationships associated with resource capture and growth. However, such trait relationships may be driven by different functions during earlier life stages. For instance, associations among regeneration traits may tell us more about plant responses to environmental cues (e.g., Donohue et al. 2010) or reliance on seed‐based resources (e.g., Kidson and Westoby 2000; Grime 1988). The need for evaluating regeneration traits, their covariation, and tradeoffs across major plant functions motivated the recently proposed seed ecological spectrum (Saatkamp et al. 2019), but more work is needed to better understand where regeneration trait variation lies within the global spectrum of plant form and function.

Germination and emergence have been repeatedly identified as the primary bottlenecks to seedling recruitment (Sharitz and McCormick 1973; Leishman and Westoby 1994; James et al. 2011; Larson et al. 2015), yet the linkage between regeneration traits and plant abundance is underexplored. Recent work with annual forbs suggests that some regeneration traits may be correlated with postrecruitment survival to adulthood in annual grasslands, suggesting an important role of regeneration traits in driving adult plant abundance (Harrison and LaForgia 2019). However, regeneration traits might not equate to adult abundance if interactions after recruitment have a disproportionate effect on survival. Ontogenetic shifts, for instance, in the types of plant interactions (e.g., change from facilitation to competition, Miriti 2006) could alter plant population dynamics. Likewise, biotic and abiotic processes that occur after emergence (e.g., pathogen attacks, Kirkpatrick and Bazzaz 1979; drought, Cook 1979) can have pronounced effects on plant mortality that may mask earlier impacts of regeneration traits on plant abundance.

Regeneration traits may also relate to adult abundance differently for native compared to exotic plant species. Some exotic populations are actively expanding, and hence recruitment success and associated traits may be more strongly linked to adult abundance than evident for long‐established native populations. In fact, recent work demonstrated that exotics generally recruited better in their introduced than their native ranges, suggesting that regeneration traits may be important for plant invasions (Pearson et al. 2022; Kožić et al. 2024). Notably, exotic species vary greatly in their success. Some invasive exotic species can attain community dominance at the expense of native species, whereas the majority of exotic species function as naturalized exotic species that remain at relatively low abundance without impacting native taxa (Ortega and Pearson 2005; Pearson et al. 2016). Traits are presumably critical for enabling the invasion of exotic plants, yet extensive research on exotic species' traits has not produced a general understanding of their link to exotic species' success (e.g., Pyšek and Richardson 2008; Van Kleunen et al. 2010; Divíšek et al. 2018; Mathakutha et al. 2019). Regeneration traits could account for some of this unexplained variation in exotic species success, in particular the degree to which exotic species are able to gain the upper hand over native species. Some exotic species may, for example, exhibit differences in germination timing to be earlier or later than natives, thereby reducing direct competition with natives for resources during early stages of growth (reviewed in Gioria et al. 2018). Similarly, seedlings of exotic species may grow faster or differentially allocate seed resources to roots or shoots to better exploit resource fluctuations relative to natives (Davis et al. 2000). Hence, invasive exotic species may have unique values for traits linked to important ecological filters, providing a simple explanation for variation in invader success (Pearson, Ortega, et al. 2018). Tying differences in native and exotic species' regeneration traits with adult abundance could improve understandings of invasion outcomes.

Here we focus on eight regeneration traits related to the seed or seedling life stage for 12 native and 12 exotic forb species, including 3 invasive exotics and 9 naturalized exotics, in semi‐arid Intermountain grasslands of western Montana, US. Our first objective was to examine associations among regeneration traits for evidence of regeneration trait syndromes or tradeoffs. We also tested whether traits and associated trait relationships differed by species status (native vs. naturalized exotics vs. invasive exotics; Objective 2). Finally, we evaluated linkages between traits and adult plant abundance, measured as cover per study species at 31 study sites spanning a broad environmental gradient, and tested for variation in trait–abundance relationships by species status (Objective 3).

## 2 Materials and Methods

### 2.1 Study System and Species

Intermountain grasslands, which occur throughout western North America, are dominated by perennial bunchgrasses and perennial forbs. This semi‐arid system receives short periods of spring rainfall, followed by hot dry summers, with another short period of rainfall in the fall. Most germination occurs during the spring or fall (Mueggler and Stewart 1980). We examined regeneration traits of 12 native and exotic plant species that varied in abundance across the study area (Table S1). For exotic species, we included both invasive exotic species with evidence of negatively impacting native species in the system and naturalized exotic species with no such evidence (based on the local‐scale correlation between abundance of the exotic and native species; Ortega and Pearson 2005; Pearson et al. 2016; Table S1). Thus, we considered *Centaurea stoebe ssp. micranthos, Linaria dalmatica*, and *Potentilla recta* invasive exotics, which typically reach high local abundance at the expense of native species, alongside naturalized exotics like *Taraxacum officinale* and *Verbascum thapsus*, which have not been shown to impact native species but are generally common components of grassland communities (Pearson et al. 2016). We note that in later research with *Veronica verna* , we found that this exotic annual behaves like other naturalized annual species and does not impact natives like other invasive exotics (Pearson et al. 2024, Figure S1). We use this updated information to classify *V. verna* as a naturalized exotic in the current study.

### 2.2 Regeneration Traits

We controlled for trait differences related to phylogeny by including at least one native and exotic species from each representative family, where possible. Furthermore, we tested for a phylogenetic signal in each trait by calculating Blomberg *K*'s using the multiPhylosignal function in the picante package (Kembel et al. 2010). The phylogenetic tree for our 24 species (Figure S1) was built using Scenario 3 in the package V.Phylomaker (Jin and Qian 2019) and shows no clear phylogenetic separation between native and exotic species included in this study. Only one of the traits had a significant phylogenetic signal (CN: *K* = 0.383, *p* = 0.041) whereas other Blomberg K's were relatively low (all *K*'s < 0.38, all *p* values > 0.1). Therefore, we did not consider any further phylogenetic corrections. Seeds for all but one species were harvested locally from bluebunch wheatgrass habitats within the Missoula valley during the summer of 2018, with seeds for each species derived from at least 50 individuals within a single population. Seeds of *Antennaria microphylla* were purchased from a local seed producer (Native Ideals Seed Company; Arlee; MT, U.S.A.). Seeds for three species needed cold stratification to break dormancy (Table S2; Appendix 1).

We measured eight regeneration traits related to germination and seedling establishment (Table S3). Regeneration traits were selected for the functional information they convey and expected ecological associations with adult abundance (see Winkler et al. 2024 for recent methodological review on assessing regeneration traits). To determine *seed mass* (SM), 15 seeds of each species were dried for 48 h at 60°C and weighed individually. Seeds for each species were germinated under standardized conditions in growth chambers (Cornelissen et al. 2003) to measure the following traits: days to germination, days to true leaf, root elongation rate, seedling mass, and relative growth rate. For these measurements, seeds of each species were divided among three petri dishes lined with filter paper (Whatman #1) and placed into growth chambers set to a diurnal setting of 12 h of light at 24°C and 12 h of dark at 13°C. The number of seeds added per petri dish varied depending on seed size and seed availability. Light levels were set to high to mimic natural conditions. Petri dishes were randomly rotated daily and watered when needed with tap water to maintain constant hydration. Seeds and seedlings were monitored at the same time daily from planting until the emergence of true leaves. Dead seedlings were removed from the study, but there were very few instances of this. Germination (presence of a protruding radicle) was recorded for individual seeds and used to determine the average *days to germination* (DTG; number of days until germination occurred) for each species. The first 12 seeds to germinate per species were transferred into the individual wells of a 12‐well plate on the day they germinated, with each well lined with one piece of filter paper (Whatman #1). Seedling roots were measured under a dissecting microscope using digital calipers to determine the root length (mm) on the day of germination. Seedlings continued to be monitored daily until the first true leaf was visible under a microscope. The date at which each seedling produced a true leaf was recorded and used to determine the *days to true leaf* (DTL) and at the same time, we remeasured seedling root length. The difference between root length on the day of germination and the day of true leaf was divided by the difference between the days to germination and days to true leaf to determine the *root elongation rate* (RER; from Larson et al. 2016). Seedlings were harvested once they produced a true leaf to standardize trait comparisons across species that might grow at different rates or experience ontogenetic trait variation (e.g., Mason et al. 2013; Garbowski et al. 2021) by this critical developmental stage that roughly marks the transition from heterotrophy to autotrophy (e.g., Steeves and Sussex 1989). After harvest, seedlings (above and belowground) were dried at 60°C for 48 h prior to weighing. *Seedling total mass* (STM) was calculated for each individual as the sum of the above and belowground dry mass. The *relative growth rate* (RGR) was calculated as STM/(days to true leaf—days to germination; Larson et al. 2016).

To determine *seedling* C:N (CN) biomass ratio and *specific leaf area* (SLA), we grew plants from seed in a greenhouse in conditions that aligned with those of the growth chamber (12 h of high light at 24°C and 12 h of darkness/lower light at 13°C). For each species, we scattered a small number of seeds onto the surface of 8 cm × 8 cm square plastic pots filled with potting soil (Fox Farm, Arcata, CA, USA). Seedlings were thinned to one plant per pot after emergence and harvested within 24 h of producing their third set of true leaves. We selected 10 seedlings for each species, scanned their first true leaves (first leaf after cotyledon) on a flatbed scanner (Epson V33), and used these photographs to measure leaf area in ImageJ (Rueden et al. 2017). Leaves of each individual were kept separate and subsequently dried at 60°C for 48 h and weighed. For each leaf, SLA was calculated by dividing leaf area by leaf mass. All remaining seedling biomass was dried at 60°C for 48 h, bulked, ground, and analyzed for percent carbon and nitrogen (Eurovector elemental analyzer, Pavia, Italy).

### 2.3 Plant Surveys and Environmental Data

We determined adult plant abundance in natural plant communities by estimating percent cover for each species present within 20 randomly located 1‐m^2^ plots in each of 31 grassland sites (620 plots) in bluebunch wheatgrass ( *Pseudoroegneria spicata* ) habitats (Mueggler and Stewart 1980) spread across a 20,000 km area of western Montana. Sites were selected to span a broad area and meet criteria for the study outlined in Pearson et al. (2016). Surveys were conducted from late May to early July in 2011, 2012, and 2014 (detailed methods in Pearson et al. 2016).

To account for environmental variability in our analyses, we quantified the environmental gradient across these communities by extracting 19 Bioclim variables from the WorldClim database (Hijmans et al. 2005) for each of our 31 study sites. Bioclim variables summarize important annual and seasonal climatic variation (see Table S4 for details); variation known to be important for seed germination, seedling recruitment, and species distributions (e.g., Leishman and Westoby 1994; Kitajima and Fenner 2000; Chesson et al. 2004). For this study, we used 30‐year averages of each variable (1970–2000) and reduced the axes of variation to create composite variables with principal component analysis (PCA) via the R package *FACTOEXTRA* (Kassambara and Mundt 2017). PC1 explained 39.6% of the total variation in Bioclim climate variables, with high scores being primarily associated with higher amounts of precipitation at greater consistency (Table S4). PC2 explained 25.5% of the variance and was heavily influenced by Bioclim variables related to temperature (Table S4). Mean annual precipitation, precipitation seasonality, and mean diurnal temperature, three of the highest loaded variables in the precipPC, varied by 210 mm, 27.6 mm, and 2.94°C across the 31 study sites, respectively. PC1 (hereafter precipPC) was used in all subsequent analyses, as it was better than PC2 at encompassing environmental variation across our study sites (Figure S2).

### 2.4 Data Analysis

All analyses were performed in R version 4.3.0 (R Core Team 2023). To examine associations among the eight regeneration traits as measured for all 24 plant species (Objective 1), we performed a PCA using mean trait values to represent each species. Mean values of seed mass, relative growth rate, and root elongation rate were log‐transformed to meet normality assumptions. Trait values were centered and scaled. The resultant PCs that explained sufficient variance to be justified (i.e., eigenvalues > 1; Kaiser 1961) are described hereafter as regeneration trait syndromes (Trait PC1, Trait PC2) since they were driven by multiple traits (as determined by their loadings) and the number and diversity of species considered were substantial (Sinnott‐Armstrong et al. 2022).

To evaluate trait/trait syndrome differences by species status (Objective 2), we created a separate generalized linear model (GLM) that treated each regeneration trait or trait syndrome as the response and included species status (native, naturalized exotic, invasive exotic) as a fixed effect (10 GLMs). Heteroscedasticity of variances and normality of errors were checked using model diagnostic plots (Crawley 2012). For all variables, we accounted for data skewness by applying the gamma family and log‐link function.

To evaluate correlations between traits/trait syndromes and plant abundance across our study sites for native, naturalized exotic, and invasive exotic species (Objective 3), we used generalized linear mixed‐effects models (GLMMs; Miller et al. 2019). We added a trace value of 0.5 to observed cover measurements to eliminate zeroes as required for the beta distribution (Damgaard and Irvine 2019) and then modeled percent cover per species and site (mean across *n* = 20 plots) as a proportion, as is appropriate for plant percent cover data (Damgaard and Irvine 2019). Three species were sampled at fewer than four study sites (CARNUT, ARNLAT, THLARV) and were excluded from these analyses, as there was insufficient data to relate their traits to environmental conditions. A separate model was constructed for each of the eight traits (to avoid multicollinearity among traits) and for each of the two regeneration trait syndromes (Trait PC1, Trait PC2) to test for linkages to abundance. Models also accounted for species status (native, naturalized exotic, invasive exotic). While we expected that abundance, as measured at the local scale, should be greater for invasive exotics than other groups given the established linkage between this metric and exotic impact status (Pearson et al. 2016), we were particularly interested in testing whether the supremacy of invasive exotics depended on regeneration traits and conversely whether trait relationships differed by species status (i.e., trait × species status interactions). Finally, to account for environmental variability across the sites and the potential for this to condition the importance of regeneration traits (i.e., trait × environment interactions), we also included the precipPC variable in models (e.g., Hahn et al. 2019). Hence, fixed effects in all models included one of the eight regeneration traits or two TraitPC axes, species status, precipPC (see Plant Surveys), and all interactions. Random effects in initial models included site, species, and precipPC × species. The precipPC × species term allowed the effect of precipitation on abundance to vary by species. However, this additional random term did not improve model fit based on AIC, so we did not include the term in final models. We also allowed the dispersion parameter to vary among species (Damgaard and Irvine 2019), which provided a better fit based on AIC and so was retained in all models. Table 1 provides a summary of terms in the model and their interpretation. All GLMMs were fit using the *glmmTMB* package in R (Brooks et al. 2017). For all models, Wald X^2^‐ and *p* values were estimated using the “Anova” function in the *CAR* package (Fox and Weisberg 2011). Model residuals were assessed using the *DHARMa* package (Hartig 2022), and post hoc contrasts were conducted with the *EMMEANS* package (Lenth 2018), when appropriate. For all analyses, we consider *p* ≤ 0.05 as significant and *p* ≤ 0.10 as marginally significant.

**TABLE 1:** Summary of models related to the interactive effects of regeneration traits (Trait), species status (SpStat), and precipitation (Precip) on abundance of 12 native and 12 exotic forbs in Intermountain grasslands of Montana, USA.

	Interpretation	SLA	DTL	RER
		χ^2^	χ^2^	χ^2^
Fixed effects				
Trait	Species' trait values affect their abundance	0.14	5.17*	0.29
SpStat	Natives, naturalized, and invasive exotics differ in their abundance	33.8*	35.3*	27.5*
Precip	Precip affects abundance	0.41	0.39	0.41
Trait × SpStat	The effect of trait values on abundance differs among natives, naturalized, and invasive exotics	7.64*	0.71	2.72
Trait × Precip	The effect of trait values on abundance varies across the precip gradient	0.02	0.04	< 0.01
SpStat × Precip	The effect of precipitation on abundance differs among natives, naturalized, and invasive exotics	7.12*	6.38*	7.29*
Trait × SpStat × Precip	The effect of trait values on abundance varies across the precip gradient differently for natives, naturalized, and invasive exotics	4.16	0.40	2.21
Random effects		***σ*** ^**2**^	***σ*** ^**2**^	***σ*** ^**2**^
Species (intercept)	Variation among species	2.1E‐01	4.4E‐01	2.6E‐01
Site (intercept)	Variation among sites	1.3E‐09	1.6E‐05	9.9E‐010

	CN	RGR	DTG	STM	SM	TraitPC1	TraitPC2
	χ^2^	χ^2^	χ^2^	χ^2^	χ^2^	χ^2^	χ^2^
Fixed effects							
Trait	2.72^**#**^	< 0.01	1.95	0.40	0.34	2.85^#^	3.65
SpStat	22.3*	28.0*	30.8*	28.9*	30.4*	31.9*	36.4*
Precip	0.38	0.42	0.40	0.41	0.40	0.40	0.38
Trait × SpStat	3.59	3.34	2.81	3.93	5.28^#^	2.54	6.17*
Trait × Precip	0.05	0.00	0.87	0.10	0.21	0.04	0.08
SpStat × Precip	5.62^#^	7.25*	6.15*	7.30*	7.24*	7.09*	7.18*
Trait × SpStat × Precip	0.43	3.01	0.48	3.35	3.50	1.68	0.80
Random effects	***σ*** ^**2**^	***σ*** ^**2**^	***σ*** ^**2**^	***σ*** ^**2**^	***σ*** ^**2**^	***σ*** ^**2**^	***σ*** ^**2**^
Species (intercept)	4.2E‐01	4.1E‐01	4.8E‐01	4.7E‐01	4.6E‐01	2.3E‐01	4.6E‐01
Site (intercept)	1.0E‐01	1.6E‐09	1.3E‐09	1.3E‐09	1.6E‐09	1.4E‐09	1.9E‐09

*Note:* The summary includes model terms, interpretation, and analysis of deviation values (wald χ^2^) for eight regeneration traits, two trait PC axes representing trait syndromes (Table S5), and 24 native, naturalized exotic, and invasive exotic plant species (Table S1). Three species (ARNLAT, CARNUT, THLARV) were excluded from these analyses due to low detection in our surveys. We used PCA to summarize the environmental variation across our study sites and included the most important PC axis, which was mainly associated with increased amount and consistency of precipitation (precipPC; Table S4) in models. Symbols denote model term significance (*p ≤ 0.05; ^#^ *p* ≤ 0.10). Trait abbreviations can be found in Table S3.

## 3 Results

### 3.1 Trait Associations, Tradeoffs, and Differences by Plant Origin and Exotic Impact

**FIGURE 1:**
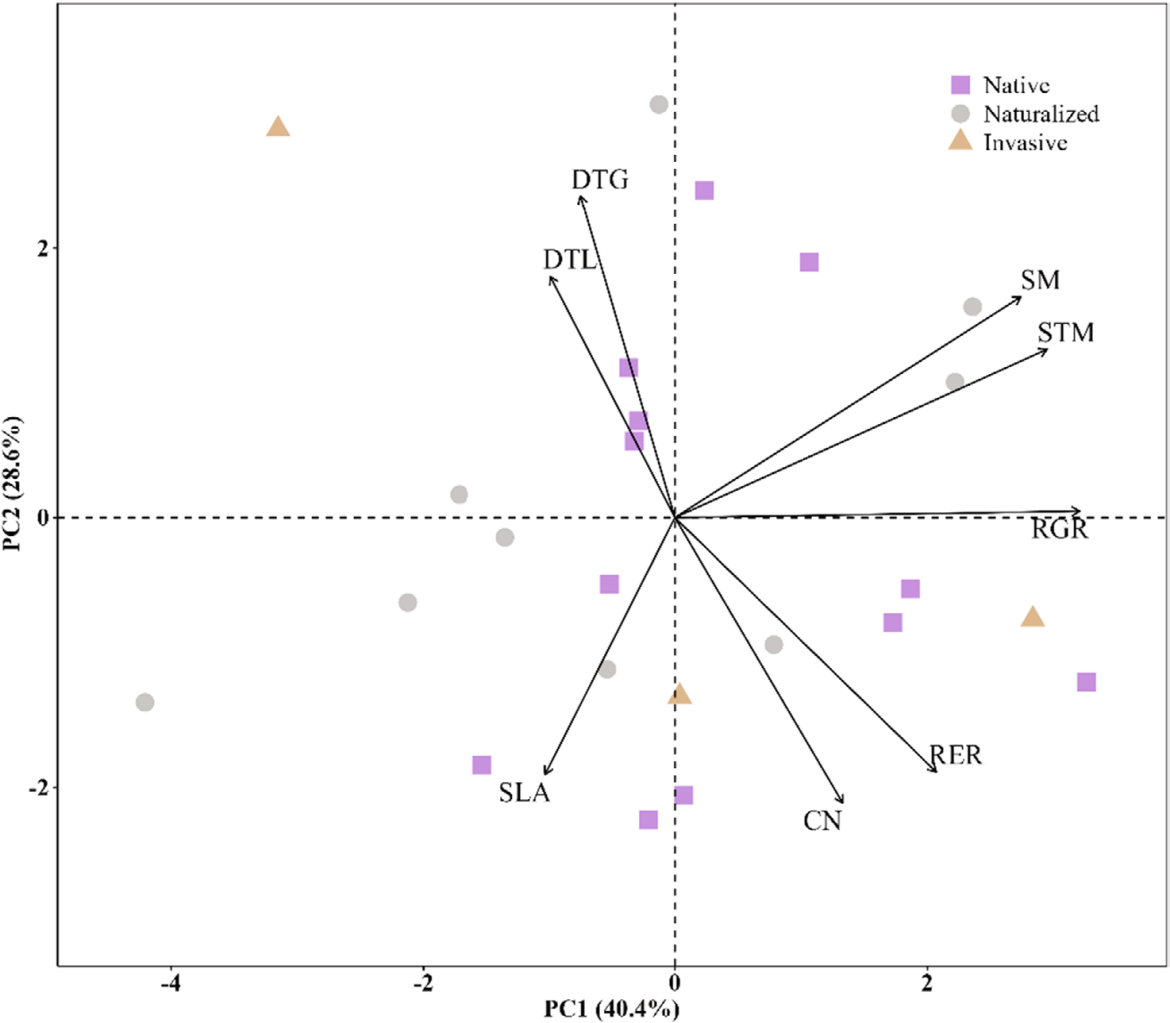
Principal components analysis (PCA) of eight regeneration traits (black arrows represent trait loadings) across 24 native, naturalized exotic, and invasive exotic forb species. PC1 (40.4% of the variance; TraitPC1) was positively associated with seed mass (SM) and seedling traits related to growth. Specifically, species with high scores for this PC had larger seeds (high SM) and produced bigger seedlings (high STM) that grew faster (high RER and RGR). PC2 (28.6% of the variance; TraitPC2) revealed a potential tradeoff between trait strategies related to development and growth (DTG, DTL, and SM) and trait strategies related to resource management (SLA, CN, and RER). High scores for this PC identified larger‐seeded (high SM) species that germinated slower (more DTG) and produced slower maturing seedlings (more DTL) with slower growing roots (low RER), thicker leaves (low SLA), and relatively high amounts of N versus C (low CN). Trait abbreviations are as follows: CN, seedling C:N; DTG, days to germination; DTL, days to true leaf; RER, root elongation rate; RGR, relative growth rate; SLA, specific leaf area; SM, seed mass; STM, seedling total mass.

Our PCA based on the eight regeneration traits as measured across 24 species of grassland forbs revealed two unique trait syndromes in this system (Figure 1; Table S5). The first principal component (TraitPC1; 40.4% of the variation) was strongly associated with seed mass and seedling traits related to growth. Specifically, species with high scores for this PC had larger seeds (high SM) and produced bigger seedlings (high STM) that grew faster (high RER and RGR).

The second principal component (TraitPC2; 28.6% of the variation; Figure 1; Table S5) revealed a tradeoff between strategies related to fast development and growth and trait strategies related to resource management (i.e., allocation, acquisition). High scores for this PC identified larger‐seeded (high SM) species that germinated slower (more DTG) and produced slower maturing seedlings (more DTL) with slower growing roots (low RER), thicker leaves (low SLA), and relatively high amounts of N versus C (low CN).

**FIGURE 2:**
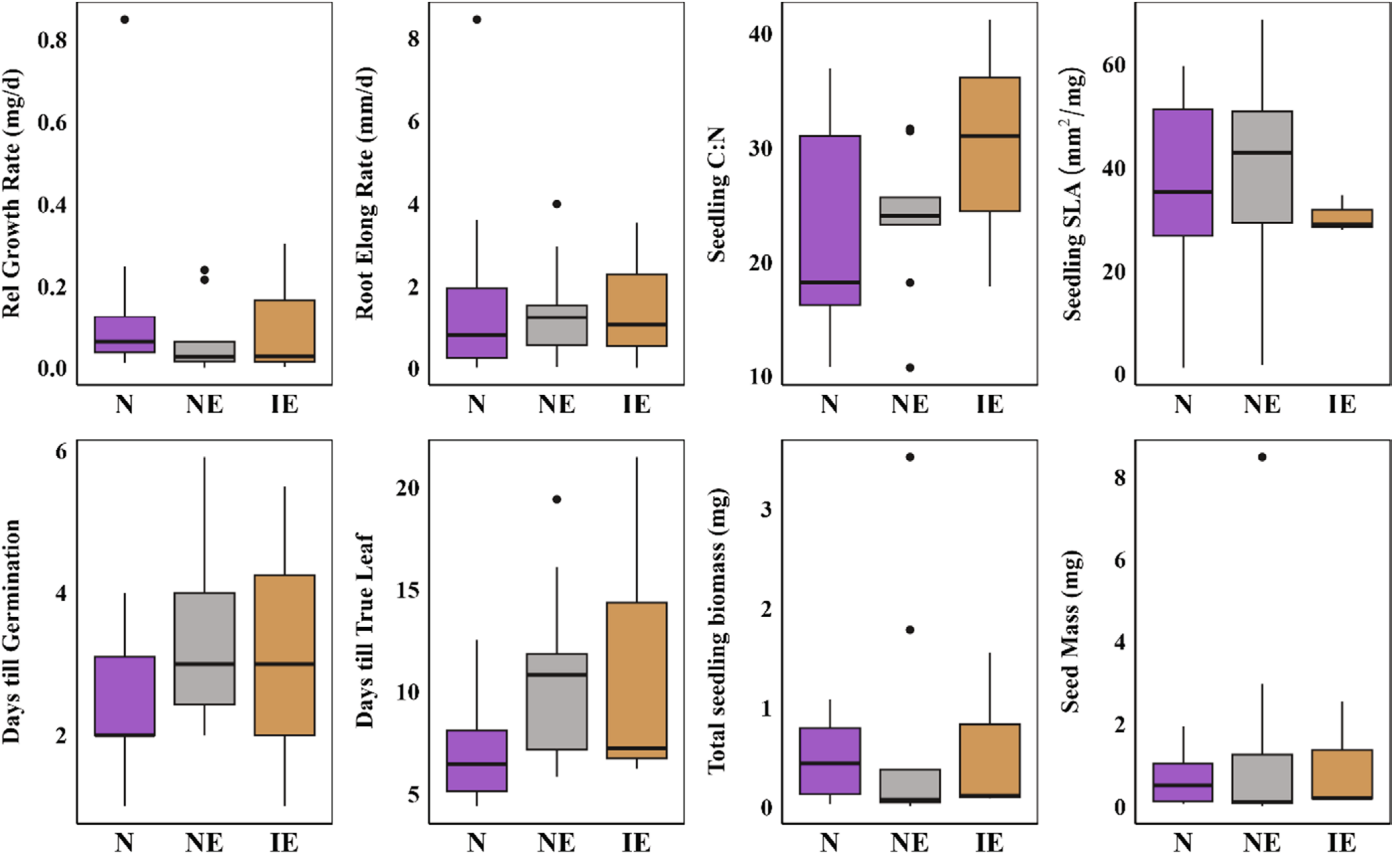
Eight regeneration traits compared among 12 native (N), nine naturalized exotic (NE), and three invasive exotic (IE) perennial forbs (see Table S6 for model results).

Neither trait values nor trait syndromes differed among species classified as natives, naturalized exotics, and invasive exotics (Table S6; Figure 2).

### 3.2 Relation of Regeneration Traits and Trait Syndromes to Adult Plant Abundance

**FIGURE 3:**
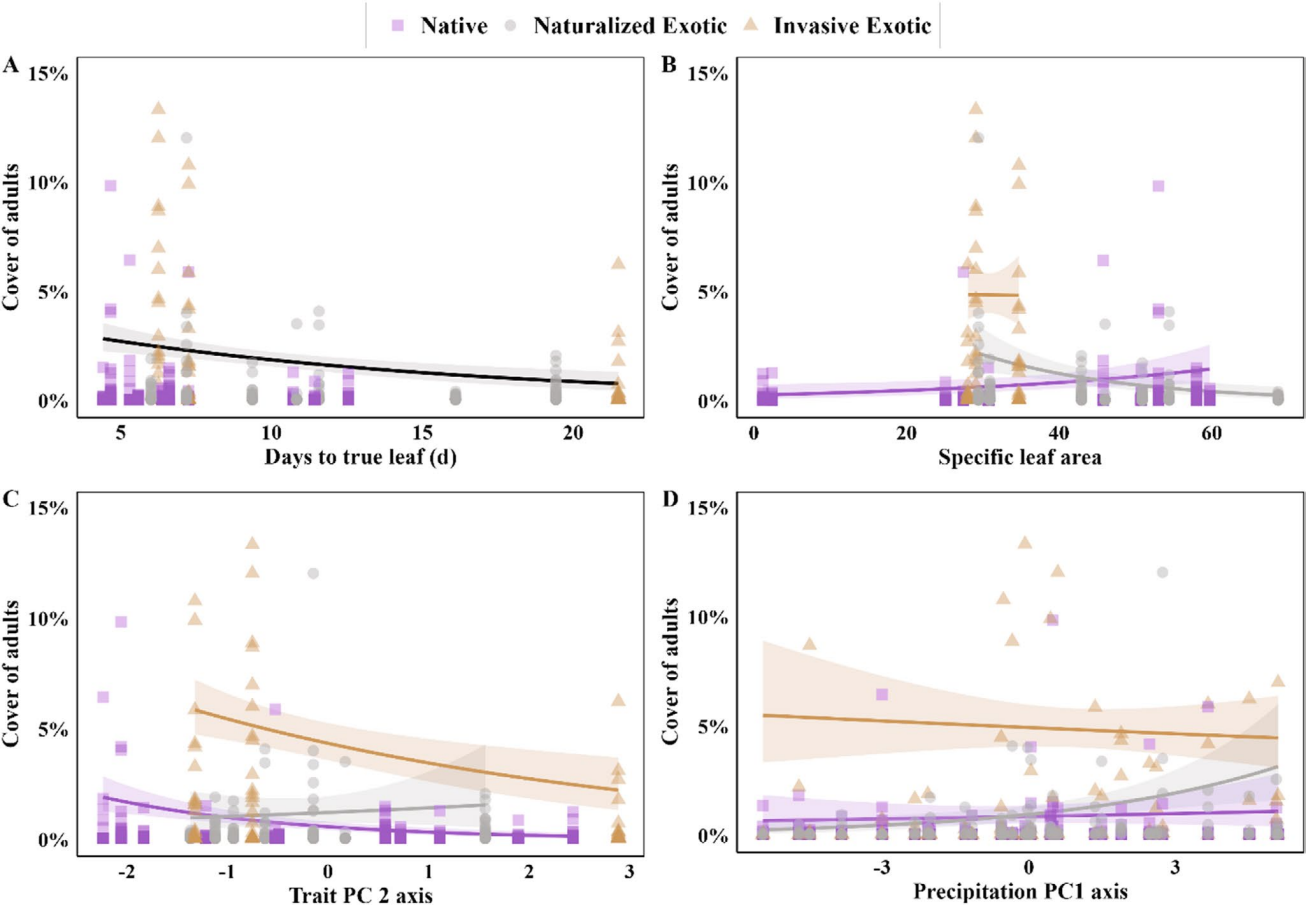
Relationships between abundance (percent cover) of 24 forb species at 31 grassland sites and the following model covariates: (A) days to true leaf, (B) specific leaf area, (C) TraitPC2, and (D) precipPC axis. Symbols differentiate species status (native, naturalized exotics, invasive exotics). Predicted relationships are shown in cases where either the covariate (black line) or covariate × species status effects (line color matching species status symbol) were significant (*p* ≤ 0.05). Each point is the mean percent cover of a single forb species averaged across 20 plots at each study site (see Table 1 for model results).

We found several individual or interacting effects of regeneration traits, species status, and precipitation on plant abundance (Table 1). For models constructed with individual traits, the relationship between traits and abundance was not strong in most cases, but days to true leaf was negatively correlated with plant abundance (slope = −0.07 [95% CI: −0.12, −0.01], *p* = 0.02; Figure 3A; Table 1). In addition, seedling CN had a marginal positive correlation with plant abundance (slope = 0.001 [95% CI: −0.04, 0.04], *p* = 0.10; Table 1). For SLA, the trait–abundance relationship varied by species status, and for seed mass, this interaction was marginal (Table 1). Specifically, SLA correlated negatively with plant abundance for naturalized exotics (slope = −0.03 [95% CI: −0.06, −0.003], *p* = 0.03), but not for natives (slope = 0.01 [95% CI: −0.003, 0.03], *p* = 0.13) or invasive exotics (slope = 0.06 [95% CI: −0.14, 0.27], *p* = 0.55; Figure 3B). Conversely, seed mass had a marginal negative correlation with plant abundance for natives (slope = −0.40 [95% CI: −0.86, 0.07], *p* = 0.10), but not for naturalized exotics (slope = −0.05 [95% CI: −0.08, 0.18], *p* = 0.48) or invasive exotics (slope = 0.45 [95% CI: −0.11, 1.02], *p* = 0.12; Table 1).

When considering trait syndromes, TraitPC1 had a marginal positive correlation with species abundance (slope = 0.12 [95% CI: −0.02, 0.25], *p* = 0.08; Table 1). This means that species with larger seeds that produced bigger seedlings and grew faster tended to be more abundant overall. Trait–abundance relationships for TraitPC2, however, varied by species status (Table 1). For natives and invasive exotics, TraitPC2 values had a negative correlation with species abundance (natives: slope = −0.19 [98% CI: −0.37, −0.003]), *p* = 0.05; (invasive exotics: slope = −0.30 [95% CI: −0.62, 0.01], *p* = 0.05), while for naturalized exotics, there was no correlation with species abundance (slope = 0.28 [95% CI: −0.10, 0.66], *p* = 0.14; Figure 3C). This means that for natives and invasive exotics, those species with smaller seeds that germinated faster and produced fast‐maturing seedlings with faster growing roots, thin leaves, and low amounts of N versus C were generally more abundant. However, even when traits and trait syndromes were accounted for, invasive exotics were more abundant than naturalized exotics and natives overall (Figure 3A–C).

The precipPC variable did not show an overall relationship with species abundance or interact with traits (precipPC × trait) to influence abundance (Table 1). However, we did find evidence for a relationship between precipPC and species abundance that varied by species status independent of traits (precipPC × Sp Stat; Figure 3A; Table 1). While naturalized exotics increased across the precipPC gradient (slope = 0.08 [95% CI: 0.01, 0.15], *p* = 0.03), neither native species nor invasive exotics showed this relationship (natives, slope = −0.01 [95% CI: −0.04, 0.01], *p* = 0.31; invasive exotics, slope = 0.02 [95% CI: −0.01, 0.06], *p* = 0.22; note that this pattern was comparable across trait models, and values reported here are from a reduced model that excluded trait effects for simplicity). However, as seen when considering trait relationships, invasive exotics remained more abundant than other groups across the precipPC gradient (Figure 3D).

## 4 Discussion

In plants, seeds and seedlings possess many traits that are distinct from adults and specifically related to key developmental transitions and resulting shifts in plant function that occur during germination, seedling growth, and seedling survival (e.g., Garbowski et al. 2021; Havrilla et al. 2021). Based on correlations among eight regeneration traits, we differentiated two main regeneration trait syndromes, which, in contrast to our expectations, were shared by grassland forb species classified as natives, naturalized exotics, and invasive exotics. Our findings also support recent work (Larson et al. 2016; Saatkamp et al. 2019; Larson et al. 2020; Slate et al. 2025) indicating that regeneration traits may lie on axes independent of those identified within resource economic spectra for adult plants (e.g., Leaf Economic Spectrum; Lambers and Poorter 1992; Reich 2014). Furthermore, our results suggest that certain regeneration traits are associated with greater adult forb abundance in our system. In some cases, regeneration trait–abundance associations varied among natives, naturalized exotics, and invasive exotics, but these differences could not explain the greater abundance of invasive exotic species across our study sites.

### 4.1 Regeneration Trait Associations, Tradeoffs, and Trait Syndromes

Variation and covariation in adult plant leaf, shoot, and root traits have identified several coordinated axes or dimensions of functional trait variation widely accepted as differentiating disparate ecological strategies for recruitment, growth, and survival (e.g., CSR plant strategy model, Grime 1988; Leaf‐height‐seed strategy, Westoby 1998; Leaf economics spectrum, Wright et al. 2004). Our results align with other recent work to suggest that some aspects of forb regeneration in Intermountain grasslands may fit into these known dimensions while other aspects may add new dimensions or enrich our functional understanding of current dimensions. In particular, we found two trait associations that conflict with well‐supported trait associations for adult plants. First, our finding that in TraitPC1 seed mass was positively associated with seedling growth rates mirrors that of other recent research on very young herbaceous seedlings (Larson et al. 2020), but contradicts the inverse pattern previously reported for older seedlings and adult plants (e.g., Marañón and Grubb 1993; Gibert et al. 2016; Larson et al. 2016). Second, our finding of a positive covariance between SLA and CN also conflicts with the widely reported negative correlation between these leaf traits (Reich et al. 1992; Grubb 1988; Poorter and Bongers 2006). It is possible that trait relationships earlier in ontogeny may have more to do with seed‐based resources than resource use strategies (Larson et al. 2020) and/or reflect the higher sensitivity that seedlings have to stressful environmental conditions than their well‐established adult counterparts (Shipley et al. 1989; Slate et al. 2025).

While understanding relationships between individual traits can be insightful, selective pressures under various evolutionary, developmental, and structural constraints can lead to multiple functional traits being tightly associated. Identification of broad trait syndromes can improve our general understanding of plant communities and simplify our ability to understand how communities will respond to environmental change (e.g., Sinnott‐Armstrong et al. 2022). We identified two main regeneration trait syndromes used by Intermountain grassland forbs and shared by native and exotic plant species. These two trait syndromes align well with the opposing competitive–stress tolerant–ruderal strategies that have been linked to grass seedlings (Larson et al. 2016) and adult life‐history strategies (Grime 1977). Under this classification, we would expect that species aligned along TraitPC1 with large seeds that produce large fast‐growing seedlings (positive loadings for seed mass, seedling total mass, root elongation rate, and relative growth rate) should be categorized as more competitive than small‐seeded species with small slow‐growing seedlings. Growth rate has been repeatedly associated with both seedling and adult plant competitive ability (Grime 1977; Pillay and Ward 2014; Reich 2014) and seedling competition can be a major determinant of seedling survival (Maron et al. 2018). In addition to a greater competitive ability, the high environmental unpredictability associated with regeneration also means that high seed mass and relative growth rate could indicate an ability to tolerate (high seed mass has been associated with greater seedling drought tolerance, Moles and Westoby 2004; Harrison and LaForgia 2019; Larson et al. 2020) or avoid (by growing fast or completing growth prior to stressful conditions) fluctuating levels of soil moisture (e.g., Shipley et al. 1989; Pearson et al. 2024). More physiological studies are needed to better understand how differences in regeneration syndromes influence seedling competitive and drought tolerance abilities.

Our TraitPC2 axis revealed a potential tradeoff of trait strategies related to development and growth with trait strategies related to resource management and suggested a separation of trait combinations used by forbs to tolerate or avoid environmental stress. Specifically, some large seeded species that germinate and mature more slowly (positive loadings) for days to germination and days to true leaf, produce seedlings with slower growing roots (root elongation rate), thicker leaves (low SLA), and higher amounts of N versus C (low CN). Slower germination often indicates a greater specificity in germination cues, suggesting that seedling survival outside of the favorable windows for germination may be less likely (i.e., Donohue et al. 2010; Larson et al. 2016). In this case, the lower SLA and seedling CN values for these species could reflect the reduced photosynthetic and biomechanical demands associated with a slower root growth rate (Poorter et al. 1990). Recent research also indicates that smaller seedlings may have higher drought survival rates than large seedlings (Funk et al. 2024). Given the fact that plants with lower SLA and higher leaf N have previously been associated with greater drought tolerance or survival (Harrison and LaForgia 2019; Xiong et al. 2022; Wright et al. 2004), the benefit of slow growth, low SLA, and low CN in seedlings could be greater drought tolerance. In contrast, smaller seeded, faster developing forb seedlings with thin, carbon‐rich leaves (high SLA and CN) and the highest root elongation rates in our study had characteristics typically associated with a more ruderal lifestyle of stress avoidance (Grime 1977; Shipley et al. 1989). Importantly, many of our study species fell in the central portion of our PCA plot, indicating that the incorporation of additional regeneration traits and the addition of more species may be required for greater insight. Further understanding of how these regeneration trait syndromes connect with environmental variation and adult functional traits will greatly improve our ability to select species assemblages that increase our success with restoration and conservation endeavors in the face of uncertain climate conditions.

### 4.2 Regeneration Trait Associations With Forb Abundance, Environmental Conditions, and Differences Among Native, Naturalized Exotic, and Invasive Exotic Species

Plants encounter a wide range of biotic and abiotic filters throughout their lifetime, making it challenging to identify clear signals regarding the impact of regeneration traits on plant abundance. However, recent research in annual grasslands (Harrison and LaForgia 2019) found that species producing seedlings with shorter roots, as well as small‐seeded species producing tall seedlings with high specific leaf area (SLA), experienced higher mortality during recent drought conditions. As a result, species with these traits become less abundant with increasing drought frequencies. In semi‐arid systems, the timing and frequency of spring rainfall is unpredictable; thus, faster maturation from emergence to true leaf could reflect an ability to better synchronize germination with favorable windows for seedling growth and minimize seedling fatality (e.g., Chesson et al. 2004; Weekley et al. 2007; Donohue et al. 2010; Gioria et al. 2018). Regeneration traits may also affect adult abundance differently in native and exotic plant species. Actively spreading exotic populations may exhibit a stronger connection between recruitment success and adult abundance compared to long‐established native populations. In the current study, we found a significant negative association between one regeneration trait (days to true leaf) and species abundance, suggesting that taxa that produced faster maturing seedlings were generally more abundant, with a parallel pattern for natives, naturalized exotics, and invasive exotics alike.

We also found one regeneration trait–abundance association (SLA), a precipPC‐abundance association, and one regeneration trait syndrome–abundance association (TraitPC2) that varied significantly by species status. For naturalized exotics, species with lower SLA values were generally more abundant than those with higher SLA values; a pattern not observed for natives and invasive exotic species. Recent research found that seedlings of annual species with lower SLA experienced decreased seedling mortality during drought (Harrison and LaForgia 2019). Indeed, most naturalized exotics in our study were short‐lived (annual or biennial; Table S1) while remaining species were largely perennial, providing a potential explanation for the negative relationship between SLA and abundance evident for naturalized exotics alone. Naturalized exotics were also found at greater abundance in our high versus low precipitation sites (Figure 3A), further suggesting a greater sensitivity to drought for naturalized exotics than native or invasive exotic species. For TraitPC2, the trait axis was negatively correlated with species abundance for natives and invasive exotics, while naturalized exotics did not share this association. Thus, native and invasive exotic species with smaller seeds and faster developing and growing seedlings with thin, carbon‐rich leaves were generally more abundant in our study sites than those with the opposite traits. These patterns suggest that species producing seedlings with “fast” regeneration strategies (lower TraitPC2 scores, i.e., Reich 2014) may be able to reach greater abundance due to their ability to avoid environmental stress compared with species with “slow” regeneration strategies, at least in some arid and semi‐arid systems (e.g., Garbowski et al. 2021; Larson et al. 2020). Given the negative impact that invasive exotics have on native species and the previously established linkage between invader impact and their local abundance in our system (Pearson et al. 2016), we expected invasive exotics to be more abundant than both naturalized exotics and natives. While this pattern was indeed apparent, we found no regeneration trait or trait syndrome that could explain the higher abundance of invasive exotics found across our study sites, that is, this group remained more abundant even when regeneration traits were accounted for. Besides additional regeneration traits, future work in this and other systems should consider regeneration trait–abundance associations by species status for grasses and shrubs and include more invasive exotic species, which could alter or reinforce the patterns found here (see Tecco et al. 2010; Larson et al. 2016). Likewise, an integrated sampling approach that incorporates occurrence data from more sources (see Fletcher Jr. et al. 2019) could bolster species detections.

Recent efforts to identify differences between native and exotic plant functional traits have been inconclusive. Results have varied drastically from finding that all trait values varied by plant origin (e.g., Van Kleunen et al. 2010), to some trait values varying by plant origin (e.g., Harrison and LaForgia 2019; Mathakutha et al. 2019; Kožić et al. 2024), to no trait values varying by plant origin (e.g., Tecco et al. 2010; Slate et al. 2025). One potential reason for this variation could be that naturalized and invasive exotics are often lumped together. Multiple studies have found that naturalized exotics tend to have similar abundances as natives while invasive exotics are the species that tend to reach higher abundances and become invasive (e.g., Firn et al. 2011; Colautti et al. 2014; Pearson et al. 2017). The fact that the invasive exotic species in our study reach higher abundances by using the same regeneration trait values and trait syndromes as natives suggests that traits not measured here may be facilitating their impact. Some of these differences may even be present at early life stages. For example, in our study system, the suppressive effects of seed predation on seedling recruitment by native rodents increases with seed size and more strongly suppresses natives than invasive exotics, because natives tend to have larger seeds (Maron et al. 2018). However, a few larger‐seeded invasive exotics may have seed defense compounds that allow them to bypass rodent seed predation (Pearson et al. 2011), suggesting that novel seed traits may help explain their success. Likewise, trait differences at earlier or later maturity stages and/or other species‐specific differences known to give invasive exotic species advantages, such as higher seed production, germinability, or germination speed (Gioria et al. 2018), novel weapons (Callaway and Ridenour 2004), evolution of increased competitive ability (Blossey and Notzold 1995), escape from natural enemies (Keane and Crawley 2002), or superior competitive ability (Vilà and Weiner 2004) could also provide an advantage and account for the differences in invasive exotic abundance found here independent of regeneration traits.

In summary, ecological filters determining community structure should first act on traits of seeds and seedlings during regeneration, then those of juveniles and subsequently adults, yet most studies focus on linkages between adult functional traits and plant abundance. Our study identifies new links between regeneration traits and grassland forb abundance that imply strong selection on early plant traits and present new opportunities for evaluating regeneration trait convergence and adaptation. While unique regeneration trait syndromes reported here generally align with the competitive–stress tolerant–ruderal spectrum of strategies reported for grass seedlings (Larson et al. 2016) and adult plants (Grime 1977), they may be poor predictors of strategies used during later life stages (Havrilla et al. 2021; but see Garbowski et al. 2021) and/or vary in their ecological functions. Gaining a deeper understanding of the selection challenges seedlings face will significantly improve our ability to connect plant regeneration to more applied aspects of plant ecology such as restoration, conservation, invasion risk assessment, and the impacts of changing climate on these efforts.

## Author Contributions

**Mandy L. Slate:** conceptualization (lead), formal analysis (equal), methodology (lead), project administration (lead), visualization (lead), writing – original draft (lead), writing – review and editing (lead). **Phil G. Hahn:** formal analysis (equal), visualization (equal), writing – original draft (equal), writing – review and editing (equal). **Yvette K. Ortega:** formal analysis (equal), visualization (equal), writing – review and editing (equal). **Marisa Mancillas:** investigation (equal), writing – review and editing (supporting). **Christoph Rosche:** formal analysis (supporting), visualization (supporting), writing – review and editing (supporting). **Dean E. Pearson:** conceptualization (lead), writing – original draft (equal), writing – review and editing (equal).

## Conflicts of Interest

The authors declare no conflicts of interest.

## Supporting information


**Table S1:** List of study species with origin, life‐history strategy, family, and species status.
**Table S2:** Seed dormancy breaking treatments.
**Table S3:** Eight regeneration traits measured and used in analyses.
**Table S4:** PCA loadings of 19 bioclim variables.
**Table S5:** Factor loadings of the eight regeneration traits from PCA.
**Table S6:** Results from GLMs testing for trait differences by species status.
**Figure S1:** Phylogeny of the study plant species.
**Figure S2:** Maps of our 31 study sites.

## Data Availability

All data is available through the Dryad repository at DOI: https://doi.org/10.5061/dryad.d2547d8db.
