# Supplementary material for: Plant Regeneration Trait Syndromes, Tradeoffs, and Linkages to Adult Abundance for Native and Exotic Grassland Plants
Source: Ecol Evol. 2025 Sep 10;15(9):e72143. doi: 10.1002/ece3.72143 (PMC12422753; doi:10.1002/ece3.72143)
Supplement: Supplementary file 1 — Table S1: List of study species with origin, life‐history strategy, family, and species status. Table S2: Seed dormancy breaking treatments. Table S3: Eight regeneration traits measured and used in analyses. Table S4: PCA loadings of 19 bioclim variables. Table S5: Factor loadings of the eight regeneration traits from PCA. Table S6: Results from GLMs testing for trait differences by species status. Figure S1: Phylogeny of the study plant species. Figure S2: Maps of our 31 study sites. [file ECE3-15-e72143-s001.docx]

**Supplemental Information for: Plant Regeneration Trait Syndromes, Tradeoffs, and Linkages to Adult Abundance for Native and Exotic Grassland Plants**

Mandy L. Slate, Phil G. Hahn, Yvette K. Ortega, Marisa Mancillas, Christoph Rosche, Dean E. Pearson

Table S1. List of species with origin, life history etc

Table S2. Seed pre-treatment

Table S3. Traits, abbreviations, and units

Table S4. Loadings bioclim

Table S5. Loadings trait PCA

Table S6. GLM results

Figure S1. Phylogeny

Figure S2. Map of study sites

**Table S1.** List of forb study species and their origin, life-history strategy, family, and species status, which distinguished native species, invasive exotics with evidence of impacting native species, and naturalized exotics with no such evidence (Pearson *et al*. 2016). Seeds for all but one species were harvested locally from within the Missoula valley, with seeds for each species derived from at least fifty individuals within a single population. Seeds of *Antennaria microphylla* were purchased from a local seed producer (Native Ideals, Arlee, MT). †This species was originally classified by Pearson *et al*. 2016 (Appendix S1, Table A1) as an invasive exotic based on impact analysis that relied on low plot-level detections. Our subsequent experimental work has shown that this species functions as a naturalized exotic and is treated as such here (Pearson *et al*. 2024).

| **Origin** | **Species** | **Life-history** | **Family** | **Species Status** |
| --- | --- | --- | --- | --- |
| Native | *Achillea millefolium* | Perennial | Asteraceae | Native |
| Native | *Antennaria microphylla* | Perennial | Asteraceae | Native |
| Native | *Arenaria congesta* | Perennial | Caryophyllaceae | Native |
| Native | *Arabis holboellii* | Perennial | Brassicaceae | Native |
| Native | *Arnica latifolia* | Perennial | Asteraceae | Native |
| Native | *Astragalus inflexus* | Perennial | Fabaceae | Native |
| Native | *Collinsia parviflora* | Perennial | Plantaginaceae | Native |
| Native | *Erigeron divergens* | Perennial | Asteraceae | Native |
| Native | *Gaillardia aristata* | Perennial | Asteraceae | Native |
| Native | *Geum triflorum* | Perennial | Rosaceae | Native |
| Native | *Heterotheca villosa* | Perennial | Asteraceae | Native |
| Native | *Plantago patagonica* | Annual | Plantaginaceae | Native |
| Exotic | *Arenaria serpyllifolia* | Annual | Caryophyllaceae | Naturalized |
| Exotic | *Carduus nutans* | Biennial | Asteraceae | Naturalized |
| Exotic | *Centaurea stoebe ssp. micranthos* | Perennial | Asteraceae | Invasive |
| Exotic | *Filago arvensis* | Annual | Asteraceae | Naturalized |
| Exotic | *Linaria dalmatica* | Perennial | Plantaginaceae | Invasive |
| Exotic | *Potentilla recta* | Perennial | Rosaceae | Invasive |
| Exotic | *Sisymbrium altissimum* | Annual/Biennial | Asteraceae | Naturalized |
| Exotic | *Taraxacum officinale* | Perennial | Asteraceae | Naturalized |
| Exotic | *Thlaspi arvense* | Annual | Brassicaceae | Naturalized |
| Exotic | *Tragopogon dubius* | Biennial | Asteraceae | Naturalized |
| Exotic | *Verbascum thapsus* | Biennial | Lamiales | Naturalized |
| Exotic | *Veronica verna* | Annual | Plantaginaceae | Naturalized† |

**Table S2.** Seeds of the three species listed here were cold treated to end dormancy. Seeds of species not listed here did not require any pre-treatment for germination. Dormancy breaking treatments followed the cited recommendations noted in parentheses.

| **Species** | **Treatment (source)** |
| --- | --- |
| *Antennaria microphylla* | 60-d cold/wet stratification (prairiemoon.com) |
| *Arnica latifolia* | 30-d cold/wet stratification (npn.rngr.net) |
| *Erigeron divergens* | 60-d cold/wet stratification (prairiemoon.com) |

**Table S3.** Eight regeneration traits measured and used in analyses.

| **Trait** | **Abbr** | **Units** |
| --- | --- | --- |
| Seed mass | SM | mg |
| Specific leaf area | SLA | mm^2^/mg |
| Seedling C:N | CN | - |
| Root elongation rate | RER | mm/d |
| Relative growth rate | RGR | mg/d |
| Seedling total mass | STM | mg |
| Days to germination | DTG | d |
| Days to true leaf | DTL | d |

**Table S4**. Loadings of 19 bioclim variables representing environmental variability across 31 study sites in relation to each of two axes from a Principal Components Analysis. Loadings are bolded when significantly correlated (*p* ≤ 0.05) with the principal component. Variables are 30-year climate averages and defined in O’Donnell and Ignizio (2012).

|  | **PC1** | **PC2** |
| --- | --- | --- |
| Variable: |  |  |
| Mean annual temperature | 0.05 | **0.43** |
| Mean diurnal temperature range | **-0.32** | 0.06 |
| Isothermality | -0.09 | -**0.34** |
| Temperature seasonality | -0.22 | **0.29** |
| Maximum temperature of warmest month | -0.13 | **0.42** |
| Minimum temperature of coldest month | 0.23 | 0.13 |
| Temperature annual range | **-0.26** | **0.25** |
| Mean temperature of wettest quarter | -0.15 | 0.19 |
| Mean temperature of driest quarter | **0.26** | 0.05 |
| Mean temperature of warmest quarter | -0.04 | **0.45** |
| Mean temperature coldest quarter | 0.19 | **0.27** |
| Mean annual precipitation | **0.32** | 0.08 |
| Precipitation of wettest month | 0.11 | 0.14 |
| Precipitation of driest month | **0.34** | 0.04 |
| Precipitation seasonality | **-0.27** | 0.04 |
| Precipitation of wettest quarter | 0.15 | 0.13 |
| Precipitation of driest quarter | **0.34** | 0.06 |
| Precipitation of warmest quarter | 0.14 | 0.07 |
| Precipitation of coldest quarter | **0.33** | 0.03 |
| Eigenvalue | 7.52 | 4.84 |
| Percentage of variance explained | 39.6 | 25.5 |
| Cumulative proportion of variance |  | 65.1 |

**Table S5**. Factor loadings of the eight regeneration traits in the first two principal components. Loadings are bolded when significantly correlated (*p* ≤ 0.05) with the principal component.

|  | **PC1** | **PC2** |
| --- | --- | --- |
| Variable: |  |  |
| Specific leaf area (SLA) | -0.17 | **-0.38** |
| Seedling C:N (CN) | 0.22 | **-0.43** |
| Seed mass (SM) | **0.46** | **0.33** |
| Seedling mass (STM) | **0.50** | 0.25 |
| Days to germination (DTG) | -0.13 | **0.48** |
| Days to true leaf (DTL) | -0.17 | **0.36** |
| Root elongation rate (RER) | **0.35** | **-0.38** |
| Relative growth rate (RGR) | **0.54** | 0.01 |
| Eigenvalue  Percentage of variation explained | 3.23  40.4 | 2.28  28.6 |
| Cumulative proportion of variance |  | 69.0 |

**Table S6.** Results from GLMs testing individual traits and trait syndromes (TraitPC1, TraitPC2) for differences by species status (native, naturalized exotics, and invasive exotics).

|  | ***χ2*** | ***p*-value** |
| --- | --- | --- |
| Seed mass | 1.457 | 0.483 |
| Specific leaf area | 0.505 | 0.777 |
| Seedling C:N | 1.485 | 0.476 |
| Root elongation rate | 0.158 | 0.924 |
| Relative growth rate | 1.070 | 0.586 |
| Seedling total mass | 0.460 | 0.795 |
| Days to germination | 3.950 | 0.139 |
| Days to true leaf | 5.672 | 0.059 |
| TraitPC1 | 1.200 | 0.549 |
| TraitPC2 | 0.381 | 0.826 |


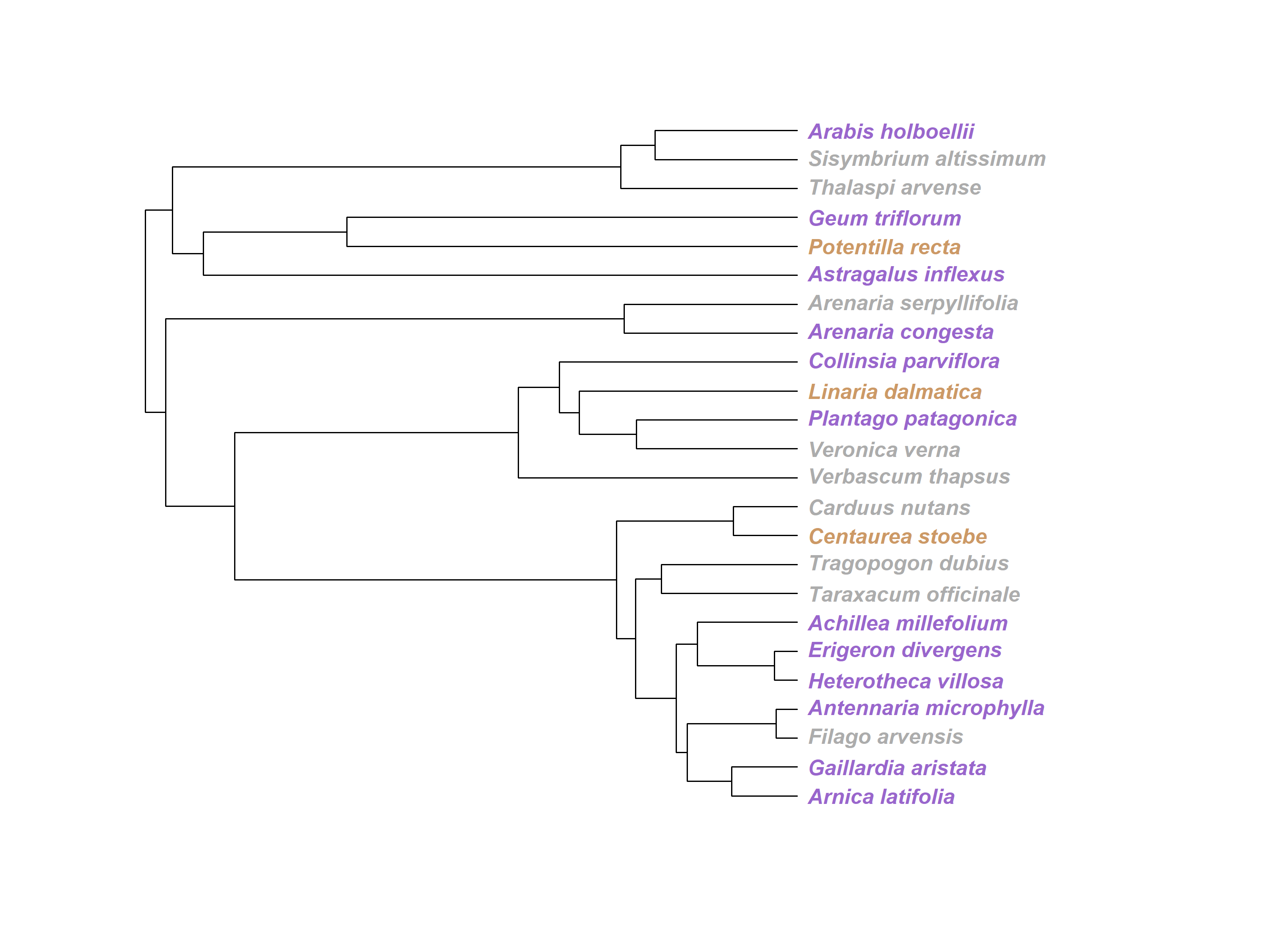


**Figure S1.** Phylogeny of the study plant species built by trimming the supertree from Zanne *et al*. (2014). Natives are purple, invasive exotics are brown and naturalized exotics are gray.


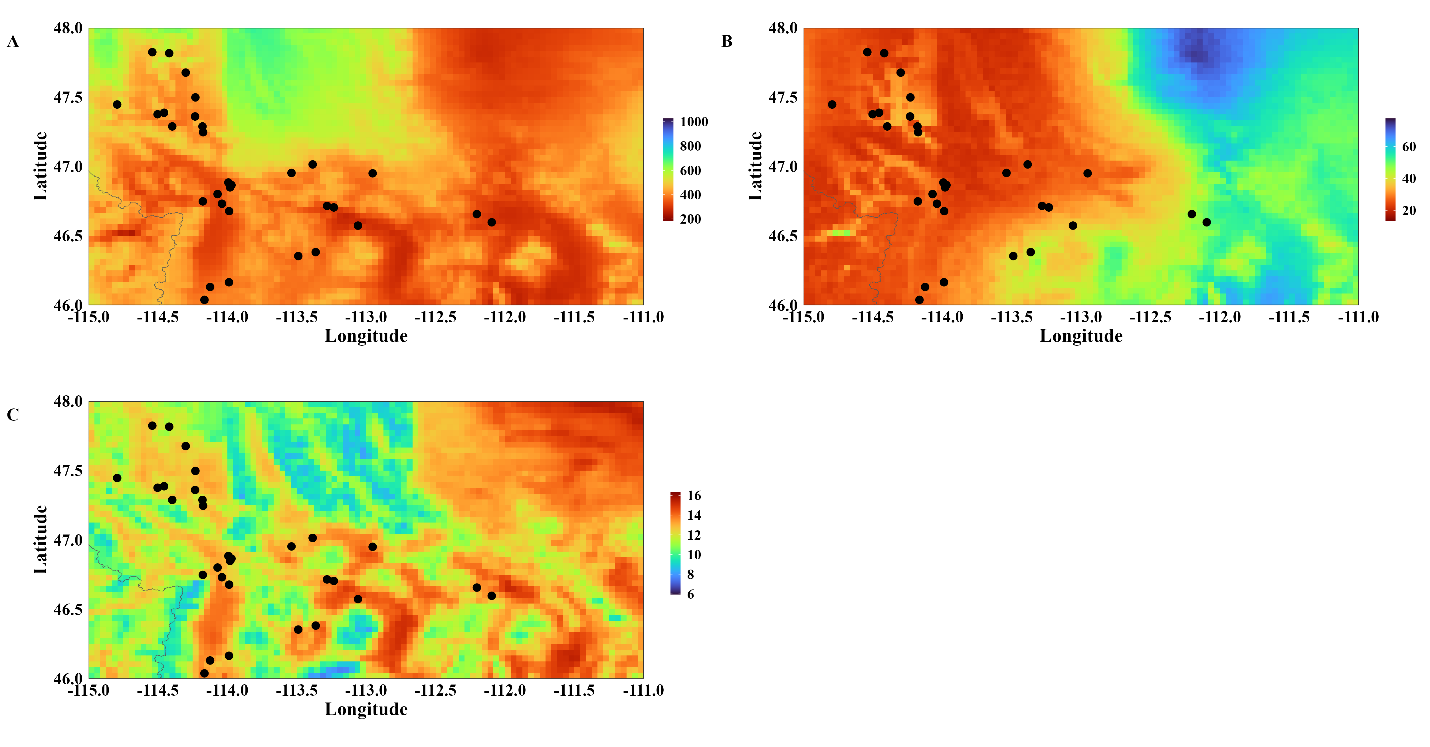


**Figure S2.** Maps of our thirty-one study sites (black dots) in western Montana, USA showing differences and range in A) mean annual precipitation (mm), B) precipitation seasonality (annual range in precipitation (coefficient of variation); mm), and C) mean diurnal temperature (ºC). These are three of the nineteen Bioclim variables used to create our environmental PC axis (precipPC; Table S4). Mean annual precipitation, precipitation seasonality, and mean diurnal temperature, three of the highest loaded variables in the precipPC, varied by 210 mm, 27.6 mm, and 2.94º C, across the 31 study sites, respectively.
